# Editorial: Insights in clinical and translational physiology: 2022

**DOI:** 10.3389/fphys.2023.1170997

**Published:** 2023-03-07

**Authors:** Yu-Sok Kim, Markus W. Hollmann, Johannes J. Van Lieshout

**Affiliations:** ^1^ Department of Internal Medicine, Medisch Centrum Leeuwarden, Amsterdam, Netherlands; ^2^ Laboratory for Clinical Cardiovascular Physiology, University of Amsterdam, Amsterdam, Netherlands; ^3^ Department of Anaesthesiology, University Medical Center (UMC), Amsterdam, Netherlands; ^4^ Department of Internal Medicine, University Medical Center (UMC), Amsterdam, Netherlands; ^5^ The David Greenfield Human Physiology Unit, National Institute for Health Research (NIHR) Nottingham Biomedical Research Centre, Pharmacology and Neuroscience, School of Life Sciences, The Medical School, University of Nottingham Medical School, Queen’s Medical Centre, Nottingham, United Kingdom

**Keywords:** type 2 diabetes, lupus nephritis, muscarinic orthosteric agonists, plasma volume, exercise, cardiac syndrome X

The aim of this editorial is to provide a background to this Frontiers Research Topic entitled “Insights in Clinical and Translational Physiology: 2022”. Experts in their respective fields of research present original data and reviews that address common diseases and conditions clinicians are meeting on an almost daily base: patients with type 2 diabetes, lupus nephritis, muscarinic orthosteric agonists (see below), the relevance of plasma volume changes during exercise for biomarkers of acute joint tissue turnover, and cardiac syndrome X. The first paper considers heart failure in patients with type 2 diabetes. Diagnosing heart failure continues to be regularly hampered by the wide variability of clinical symptoms and signs, and the diversity of possible aetiologies add to this clinical problem (Coronel et al., 2001). The peroxisome proliferator-activated receptor *γ* coactivator 1 alpha (PGC1α) is involved in the metabolic adaptation required to maintain the heart’s contractility (Oka et al., 2020). Expression of PGC1α in contracting skeletal muscle by exercise stimulates an increase in expression of fibronectin type III domain-containing protein 5 (Fndc5). This membrane protein is subsequently cleaved and secreted as irisin, a myokine, with a positive effect on energy expenditure (Bostrom et al., 2012; Villarroya, 2012). A new role for serum irisin level in patients with heart failure has been suggested (Silvestrini et al., 2019). Berezin et al. follow up by addressing the discriminatory value of serum irisin level in predicting clinical outcome in a cohort of patients type 2 diabetes mellitus (T2DM) who presented with heart failure. In these patients irisin levels were lower; adding irisin to N-terminal pro–B-type natriuretic peptide (NT-proBNP) improved the discriminative value of the cardiovascular composite.

The next paper addresses Lupus nephritis (LN), a disease with considerable mortality (Faurschou et al., 2010; Reppe Moe et al., 2019). In a recent review on LN entitled ‘*A historical appraisal of how a skin lesion became a kidney disease*’, the term ‘lupus’ was explicated as being derived from Latin for wolf (Musa et al., 2021). Originally it was introduced in the Middle Ages to denote erosive skin lesions resembling wolf bites (Airy & Eknoyan, 2019). In 1872 the Viennese dermatologist Moriz Kaposi subdivided lupus into a discoid and a systemic form and developed the concept of lupus as a systemic rather than an isolated skin disease with a potentially fatal outcome (Kaposi, 1872). Six classes of LN are distinguished of which class 3 and 4 are associated with an increased risk for the development of end-stage renal disease. In patients with LN, a high serum creatinine level at disease onset is the most commonly reported independent predictor for development of end-stage renal disease. Hitherto LN is treated with immunosuppressive agents on guidance of the LN class, based on histoimmunopathology of a renal biopsy specimen. LN is considered as a polygenic phenomenon that is more often observed in first-degree relatives of patients suffering from systemic lupus erythematosus with an estimated familial prevalence of ∼ 10–12% (Musa et al., 2021). In this topic Xu et al. move our understanding on LN forward by addressing the present insight concerning the interaction between micro RNA’s and epigenetic regulation of LN in humans.

This is followed by a study on the relevance of plasma volume changes during exercise for biomarkers of acute joint tissue turnover. When humans stand up, downward blood pooling with a fall in venous return to the heart results in a diminution of central blood volume and a consequent decrease in cardiac filling pressure and in stroke volume (Harms et al., 2020). When exercising in the upright position plasma volume does change further. Bjerre-Bastos et al. considered that when investigating plasma constituents, e.g., during or shortly following exercise, as in studies of acute joint tissue turnover, adjustment for potential plasma volume (PV) fluctuations may be required. To that purpose they studied the role of adrenergic activation in the PV changes that take place during physical activity. They compared the effects of cycling and running exercise versus adrenergic circulatory stress as a mimicry of (passive) cardiovascular exercise on changes in PV in circulatory healthy subjects with knee osteoarthritis. The primary finding is that PV decreases in response to moderate-high intensity running and cycling, and adrenaline infusion mimicked these PV changes which was interpreted as to suggest a separate influence of autonomic control on blood volume homeostasis. When reporting plasma/serum constituents measured during exercise the dynamic character of PV changes could be relevant.

The next manuscript reviews muscarinic orthosteric agonists, where the adjective ‘orthosteric’ stands for the primary, unmodulated binding site on a ligand receptor. Otto Loewi’s observation in 1921 of a reduction of heart rate by a substance that was later identified as acetylcholine activity (Ach) by Henry Dale has become the start of many discoveries on Ach function, ranging from neuromuscular junction transmission and autonomic function in the peripheral nervous system to centrally mediated cognitive processes. Ach binds to G protein-coupled muscarinic Ach receptors, and ligand-gated nicotinic Ach receptors (Shoaib & Wallace, 2020). Specifically, cholinergic (Ach-mediated) transmission that activates muscarinic receptors occurs mainly at autonomic ganglia, organs innervated by the parasympathetic division of the autonomic nervous system and in the central nervous system (Shoaib & Wallace, 2020). In 1936 Sir Henry Dale (UK) and Professor Otto Loewi (Austria) shared the Nobel Prize in Physiology or Medicine *“for their discoveries relating to the chemical transmission of nerve impulses”* (Tansey, 2006). Myslivecek calls for attention of interested researchers not deeply focused on muscarinic acetylcholine receptor (mAChR) pharmacology involved in central nervous system function studies by elaborating on the multitargeting nature of muscarinic orthosteric agonists and antagonists into more detail.

In the final paper cardiac syndrome X and uric acid blood level as its potential biomarker is considered. Cardiac syndrome X usually presents with angina pectoris (-like) symptoms and concordant electrocardiographic abnormalities reflecting metabolic cardiac stress as visualized by reversible perfusion defects on nuclear imaging but with a normal coronary angiogram. This is why this syndrome, being attributed to endothelial dysfunction, has been designated as microvascular angina (Crea & Lanza, 2004). The pathogenesis of elevated uric acid levels in patients with cardiac syndrome X (CSX) is unclear, and the results presented in recent papers on uric acid levels in patients with CSX are not univocal. As a background, uric acid, discovered in the 18^th^ century by the Swedish chemist Carl Wilhelm Scheele, is the final product of purine catabolism (Galassi & Borghi, 2015) and elevation of uric acid blood level is associated with cardiovascular disease. Harvey Kemp Jr et al. (1973) coined the term cardiac syndrome X to anginal chest pain with no evidence of significant coronary vascular abnormalities visualized on angiography. Women and men have different patterns of ischemic heart disease throughout their lifetime (Maas, 2017). In women microvascular angina is a worrisome source of chest pain hitherto largely misdiagnosed because of absence of coronary artery disease on angiography, and in some aspects this disease entity resembles syndrome X. Zu et al. analyzed the relationship between uric acid blood level and cardiac syndrome X. Here they report the results of a meta-analysis of historical groups of patients. Patients with syndrome X presented with elevated UA levels which appeared not only associated with obesity, but also with those who are not obese.

The contributions to this Clinical and Translational Physiology Research Topic are far from being focussed on one single (patho-) physiological phenomenon or disease in humans. Instead, it demands attention to the diversity of mechanisms involved in common diseases in humans as exemplified in this Journal dedicated to clinical and translational (patho-) physiology.
